# Histomorphometric evaluation of calcium phosphate bone grafts on bone repair

**DOI:** 10.1590/S1808-86942011000400007

**Published:** 2015-10-19

**Authors:** Karis Barbosa Guimarães, Belmiro Cavalcanti do Egito Vasconcelos, Francisco de Assis Limeira Júnior, Frederico Barbosa de Sousa, Emanuel Sávio de Souza Andrade, Ricardo José de Holanda Vasconcellos

**Affiliations:** 1 MSc in Dentistry - PUCRS; Graduate Student in Dentistry - University of Pernambuco in Maxillofacial Surgery and Traumatology, PhD. FOP/UPE; Assistant Professor of the Health and Education Center - Federal University of Grande - CES/UFCG; 2 PhD; Adjunct Professor and Coordinator - Graduate Program in Dentistry - University of Pernambuco. Maxillofacial surgery and Traumatology - FOP/UPE; 3 PhD. Adjunct Professor of the Graduate Program in Dentistry - Federal University of Paraíba - Oral Diagnosis - UFPB; 4 PhD. Adjunct Professor of the Graduate Program in Dentistry - Federal University of Paraíba - Oral Diagnosis - UFPB; 5 PhD; Adjunct Professor of the Graduate Program in Dentistry - University of Pernambuco, in Maxillofacial Surgery and Trauma - FOP/UPE; 6 PhD; Adjunct Professor of the Graduate Program in Dentistry - University of Pernambuco, in Maxillofacial Surgery and Trauma - FOP/UPE

**Keywords:** biocompatible materials, bone regeneration, calcium phosphates, durapatite, osteogenesis

## Abstract

**Abstract:**

Because of its biocompatibility and osteoconductive properties, calcium-phosphate cement has been used as bone surrogate.

**Objective:**

The purpose of this study was to do a histomorphometric comparison of bone regeneration using hydroxyapatite biphasic ceramic, calcium-phosphate cement and autogenous bone graft.

**Study Design:**

Prospective and laboratorial experiment.

**Materials and Methods:**

Two 5mm in diameter cavities (left and right - test and control) were made in the parietal bone of 72 rats. The GI, GII, GIII and GIV test cavities were filled with calcium-phosphate cement, biphasic ceramic hydroxyapatite, autogenous bone graft and blood. The animals were killed at 30, 60 and 90 days and the specimens underwent histomorphometric analysis.

**Results:**

The results showed that autogenous bone graft treated defects had significantly more new bone at 30 days compared to other test groups. Within 60 and 90 days, bone formation was more significant in the test groups GI, GII and GIII; GI and GII encompassed larger areas. Throughout the evaluation periods, GII tests showed more bone formation when compared to GI.

**Conclusion:**

Biomaterials depicted a significantly increase in bone content, when compared to autogenous bone graft, concerning bone regeneration.

## INTRODUCTION

Bone loss remains as one of the main problems within medical and dental specialties, very likely associated with the exposure of bone tissue to different physiological and pathological situations. Bone tissue has an impressive regenerative capacity and, in many situations, it is perfectly capable of reestablishing its architectural and mechanical properties by means of a complex process which involves local and systemic actions. Nonetheless, the bone regenerative capacity has limitations and may also fail, resulting in usually bone defects which are very large to be spontaneously filled in and repairing itself^1^.

Bone loss caused by fractures, extensive surgery and disease processes which involve the stomatognathic process, such as osteomyelitis, cystic lesions, dental tumors and periodontal bone defects, besides the continuous and growing need for osteointegration of bone grafts and implants in the receiving and donor sites, have led many researchers to develop new techniques aiming at helping bone tissue repair or accelerate the bone healing process^2,^^3^.

In order to install, recover and/or keep bone quality and volume in regions which lost their anatomical shape, different studies have tried to develop or improve new and promising biocompatible materials with osteoconduction properties, which promote bone defect repair^4^, ^5^, ^6^, ^7^. In order to do that, these materials must comply with some conditions: support must be made of a bioactive or bio inert material; its shape and size must promote bone growth from inside and bone deposition must happen by replacement^8^. Bone conduction materials are unable to induce cell differentiation of osteoblasts, nonetheless, they fill out the defect, guiding the new cells originated by osteoprogenitor cell proliferation, coming from the margins of the defect, as they promote bone neogenesis^5,^^6,^^7,^^9,^^10^.

Having in mind the larger material bioavailability, morbidity reduction, less surgical time, a greater facility to execute the surgery - provided by the filling up of osteoconduction material; the goal of the present paper is to investigate, through morphometric analysis, the process of bone regeneration, carefully measuring the bone malformation, influenced by calcium phosphate cement, biphasic hydroxyapatite ceramic and by the autogenous bone graft in the repair process of critical bone defects, made to the skull of Wistar lineage rats.

## MATERIALS AND METHODS

This was a prospective, lab experimental, paired and blind study, with randomly assessed work groups. The animal model was the male *Wistar Albinus*, rat, weighing between 350 and 400 grams, clinically healthy and making up a total sample of 72 rats, kept under natural conditions of light, moisture and temperature at the animal housing facility of the Federal University of Campina Grande (Campina Grande/Brazil). During the entire experimental period, the animals were fed a standard solid diet and ad libitum water. The animals were broken down into four groups, and each group was further divided into three groups, according to observation periods of 30, 60 and 90 days.

The present study's protocol was submitted to approval by the Ethics in Research with Animals Committee, under protocol # 002/09.

Under general anesthesia (Ketamine Chloridrate at 10% (0.05 ml/100g) and Xylazine Chloridrate at 2% (0.025 ml/100g), the animals were submitted to antisepsis on the site chose for the surgical intervention, the craniofacial region, extending side-to-side, between the external ears, and anteroposteriorly, the regions of the nasal and occipital bones. This way, we made a linear coronal incision, on the skin and the subcutaneous tissue, of approximately 1.5 cm in extension with a complete posterior mucoperiosteal shifting until the complete exposure of both parietal bones of the animal. Two standardized 5mm bone defects were made to the side of the median sagittal suture, with a 4mm distance between them, measured by a surgical caliper and having as a depth parameter, the bone cortical breakage and exposure, without perforation, of the dura mater. The left-side bone defect was made more cranially, while the contralateral one was more caudal, aiming at reducing the likelihood of bone filling material shifting from one bone defect to the other at the time of tissue suturing.

After making both bone cavities, only the left-side bone cavities were filled out by the biomaterial in the external cortical bone, while the right-side bone cavities did not receive any filling material, only the blood clot from the animal - acting as the control group in the study when compared to the filling material and the autogenous bone graft. Animals from groups I, II, III and IV had their left-side cavities filled out by calcium phosphate (Norian CRS *Fast Set Putty* - Synthes North America Products, California, United States), by biphasic hydroxyapatite ceramic (made up of 40% of hydroxyapatite and 60% of calcium phosphate cement) (Genius, Baumer S.A., Campo Largo, Paraná, Brazil), and by autogenous bone graft and the animal's own blood clot.

The calcium phosphate cement was prepared according to the manufacturer's instruction, mixing the two components: the calcium phosphate as a sterile powder (0.25g) and a sterile solvent based on sodium phosphate, which made a homogeneous and uniform paste of calcium phosphate cement. The biphasic hydroxyapatite ceramic (0.25g), because of its microgranular nature, was mixed to a small amount of blood obtained from the animal at the time of the incision or after the osteotomy needed to form the bone defects. The autogenous bone graft was obtained from the circular segment of the skull, removed during the bone defect making. For such, the circular segment was divided in four smaller pieces, and three were autogenous bone fragments inserted into the left bone cavity.

After making it and the proper filling up of the test cavity with the biomaterial, a non-resorbable membrane was placed (Gen-Derm®, Baumer S.A., Campo Largo, Paraná, Brazil) on both the bone cavities made, with the intent of avoiding excessive pressure and connective tissue invasion of the experimental and control cavities. This membrane was cut and fit passively on the rat's skull, without the need to fix it. After membrane insertion and fitting, the periosteum, the muscle tissue and the skin of the entire animal were repositioned and sutured with nylon monofilament wire (4-0).

After the observation periods, the animals were slaughtered at 30, 60 and 90 days after surgery, through intracardiac administration of potassium chloride, after general anesthesia, according to the protocol described at the time of the surgery technique, until we noticed the termination of vital signs. The skulls from each animal were removed, making up the surgical specimens from each group. The specimens obtained were dip in 10% buffered formalin solution for 02 days, they were later decalcified in a 5% nitric oxide solution and routinely processed by the hematoxylin-eosin technique, according to the protocol established by the buccal pathology lab of the Dentistry School of the University of Pernambuco.

The slides were studied under light microscopy. The slides from each animal were submitted to microscopic exam through the capture system, under 20x magnification and image analyses by a set of *pixels* - Image-Pro Plus^U+000AE^. After image capture in *JPEG* format, they were sent to the Image Tool Scripting Language^U+000AE^ - histomorphometry software. Through this histomorphometric software we measured the desired areas, without prior knowledge concerning image distribution in their respective study groups, by outlining the desired regions with the mouse, thus assessing the bone repair development by measuring the bone neogenesis areas. The values obtained were submitted to descriptive and inferential uni and bivariate statistical analyses by means of the SPSS for Windows - version 15 software, using the Mann-Whitney U test (*p*≤ 0.05).

## RESULTS

**Chart 1:**
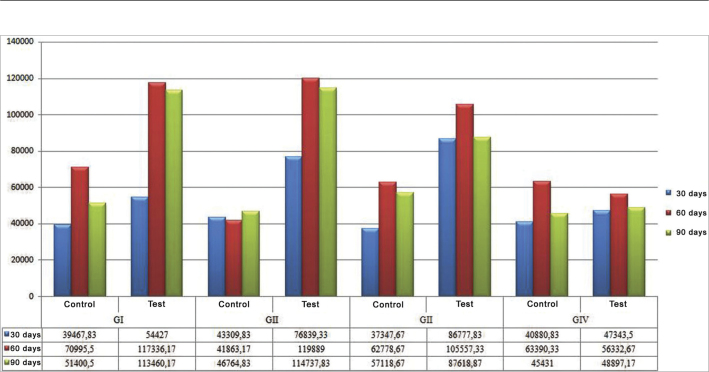
Control and test groups' histomorphometric evaluation by assessment of time and according to the material tested. - Blue: 30 days, Red: 60 days, Green: 90 days.

**Chart 2:**
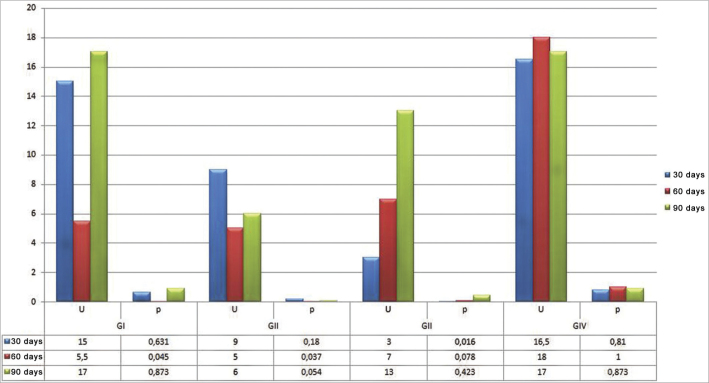
Histomorphometric assessment according to the test and control groups' mean values, by evaluation time and according to the material tested. Blue: 30 days; Red: 60 days; and Green: 90 days.

Chart 1, Chart 2 depict the mean values concerning the newly created trabeculate bone areas, quantitatively analyzed in each group, as well as the observation periods analyzed from a set of pixels. The histomorphometric assessment was calculated a priori, by comparing the groups individually by tested material. Among all the paired comparisons, the only one which proved significant was the test group after 60 days of observation (*p*=0.000). Mann-Whitney U test of paired comparisons showed that the difference is in the control group when compared to the others, it has a lower histomorphometric value when compared to the other materials tested, and throughout all the observation periods tested.

Histomorphometric results from the total areas of the newly created bone trabeculate showed that the groups submitted to bone filling biomaterial (osteoconduction and autogenous bone graft) showed bone trabeculate area values higher than the control group of the same animal and of the experiment's negative control group (Group IV); however, without statistically significant association between the groups. Analyzing total osteogenesis, according to the different observation periods, we noticed homogeneous bone neogenesis between the test and control groups at 60 and 90 days. The increase in osteogenesis seen during the transition from the 30 to 60 days of observation and the osteogenesis maintenance from 60 to 90 days of observation must be stressed in all the test groups submitted to osteoconduction material insertion (calcium phosphate cement and biphasic hydroxyapatite ceramic) and the autogenous bone graft; however, with a greater intensity in the groups submitted to bone filling biomaterials (Charts 1 and 2). It is important to stress the drop in osteogenesis that happened during the transition from 60 to 90 days of observation, in the test cavities, concerning all test materials.

As we quantitatively analyze the newly created trabeculate bone from each tested material, its respective control and test cavities, we noticed statistically significant differences in the group submitted to autogenous bone graft at 30 days (*p*=0.016), and the histomorphometric value was higher in the test cavity. The second statistically significant difference was in the calcium phosphate cement group at 60 days (*p*=0.045), being higher in the bone trabeculate areas in the test cavities and the third statistically significant difference was in the biphasic hydroxyapatite ceramic group at 60 days (*p*=0.037), also showing higher bone neogenesis values in the test group. The values associated with the test are depicted on Table 1; nonetheless, the mean values corresponding to these groups may be seen in the previous charts.

**Table 1:** Test and control groups' histomorphometric assessment by assessment time and according to the tested material.

Group	Assessment time (in days)	GI	GII	GIII	GIV
	30 and 60	*p*=0,337	*p*=0,936	*p*=0,172	*p*=0,628
Control	60 and 90	*p*=0,337	*p*=0,872	*p*=0,631	*p*=0,872
	30 and 90	*p*=0,631	*p*=0,747	*p*=0,423	*p*=0,810
	30 and 60	*p*=0,016*	*p*=0,337	*p*=0,521	*p*=0,631
Test	60 and 90	*p*=0,337	*p*=0,873	*p*=0,423	*p*=0,749
	30 and 90	*p*=0,936	*p*=0,016*	*p*=0,748	*p*=1,000

(*p*) Statistical association

Source: Research data, 2010.

(*) has statistically significant difference for *p*≤0.05.

When we paired the assessment times with the control and test cavities of the materials tested, two situations were considered statistically significant. The first was seen in the transition between 30 and 60 days in the calcium phosphate cement group test cavity, in which the mean value of the newly formed bone trabeculate was higher at 60 days; the second statistically significant difference was seen in the test cavity of the biphasic hydroxyapatite ceramic group during the 30 to 90 day combination period, and the histomorphometric mean values were higher at 90 days (Table 1 for inference tests and Charts 1 and 2 for descriptive statistics).

## DISCUSSION

Numerous biomaterials are constantly being studied in order to find one to replace bone tissue, so as to provide a feasible alternative for the autogenous bone graft which, although being the best and better accepted bone substitute - for having essential characteristics of osteoinduction, osteoconduction and osteoprogenicity^4,^^11,^^12^, it has disadvantages, especially considering morbidity because of the need for bone removal from the donor area and its insertion in the receiving area, thus increasing the number of surgical interventions, besides the amount of bone graft available, especially in cases of intraoral donor areas^13^, ^14^, ^15^. We also noticed different degrees of post-op bone resorption, changing the previously obtained results^16^.

The biomaterial used as bone surrogate must be biocompatible, atoxic, resist deformation, resorption resistant or not according to the decided application and, should they be resorbable, they must be metabolized by the body or excreted through normal physiological pathways^35^-^7,^^14,^^15^.

The calcium phosphate bioceramic continue being studied and utilized in tissue reconstruction procedures, having seen that their physicochemical characteristics and properties are able to induce a favorable biological response. The calcium phosphate bioceramics have biocompatibility, bioactivity and osteoconduction properties, which shows that as they are inserted in the receiving bed, they do not cause immune responses, they are able to connect directly to the bone tissue and enable bone growth throughout its surface^4,^^17^. The characteristics seen in both bone filling materials, the biphasic hydroxyapatite ceramic (Gen-Phos® HA TCP, Genius, Baumer, Inc.) and the calcium phosphate cement (Norian CRS®, Synthes GmbH *&* CO, KG), corroborate the conceptually necessary properties for an ideal biomaterial, since they do not cause exacerbated inflammatory reaction, were not encapsulated or rejected by the body and enabled the osteoprogenitor cells adjacent to the bone defect to differentiate through the framework generated by such materials, proving osteoconduction^5,^^6,^^7,^^9,^^10^.

The methodology employed in the present study is in agreement with the one employed by numerous studies aiming at assessing the behavior of bone filling materials^18^, ^19^, ^20^, ^21^. Since the sample subject chosen were young male adult wistar rats, the sample inclusion criterion aimed at avoiding the interference of hormonal factors and guarantee increased physiological and metabolic conditions in young rodents.

Rats have been used as experimental models in order to assess bone scaring. Thus, the scar response has been documented under a variety of conditions. Rats' laminar and long bones have a layer of densely mineralized and well-formed cortical bone, which do not suffer internal remodeling and, consequently, are very fit to bone kinetics studies. Prior studies have been reported in a six-day alveolar bone remodeling for rats, compared at 60 and 120 days for human adults^22^.

As surgical sites, we chose to make the two circular defects laterally to the median sagittal suture, in the rats' skulls. The circular defect diameter was 5mm, which was the diameter considered critical for bone regeneration^23,^^24^. According to Schmitz & Hollinger^25^, the critical defect has an area which prevents spontaneous bone regeneration, unless another osteogenic, osteoconduction or osteoinduction material is inserted inside or over the bone defect. As to the creation of two bone cavities in the same animal, the present paper was based on the studies carried out by Bosch et al.^23^, Grandi^18^ and León^21^, which stated that the creation of two spherical cavities on the rats' skulls create a paired study model, proper to assess the efficacy of the osteogenesis promoter materials used to stimulate bone regeneration.

According to the data obtained with bone regeneration, it is not possible to assess whether the 5mm defect behaves as critical, having seen that there was bone neogenesis in the group which did not receive bone filling material, receiving only blood clot in the bone defect (control group of the test animals and negative controls). The bone regeneration seen in the control groups (control group of test animals and negative controls), is different from those in the studies by Bosch et al.^23^, as well as those from Donos et al.^24^, which proved that the 5mm defects on the rats' skulls would not promote any spontaneous bone regeneration sign after 30 or 60 days of observation.

It is necessary to consider the anatomical relation which exists between the parietal bone morphology, bone site chosen for the bone defect, and the bone regeneration process developed along the observation periods. It is known that the parietal bone is our laminar bone, predominantly cortical and with little medullary compo-nent^26^. As we created the bone defect, it involved the bone's internal and external corticals, without perforating the meninges, especially the dura mater. From then, we observe that the only direction of the osteogenesis would be the one induced from the borders of the bone defect on the skull^24,^^27^. It is also known that the external and internal corticals from the laminar bones are internally and externally coated by connective membrane layers, called periosteum and endosteum^26,^^27^. Thus, it was suggested that the bone regeneration process presented by the negative control group (GIV) could be favored by the differentiation of the osteogenic cells found in the periosteum, repositioned at the time of the wound closure, as well as the endosteum, preserved in the vicinities of the defect borders, as per advocated by Dângelo & Fattini^26^ and Takeuchi et al.^27^.

The histomorphometric analyses we made enabled us to detect the presence of bone in the defect and to quantify the size of the newly formed area. The importance of this type of analysis has been confirmed in the studies by Ferreira da Silva^20^, Cavalcanti et al.^28^, Grandi^18^ and León^21^, who reported that quantitative analysis was essential for studies which aimed at assessing the effectiveness of new treatment modalities concerning bone neogenesis.

Upon observing bone neogenesis, from the quantitative viewpoint, the results obtained from this experiment show differences between the control and test groups, concerning the different bone filling material utilized, as well as between these groups and the negative control group. Larger bone trabeculae areas were seen in the test cavities of the groups submitted to bone filling material; however, without statistically significant association at 30 and 90 days, which showed the biphasic hydroxyapatite ceramic, calcium phosphate cement and autogenous bone graft positive action on the bone repair process. These results are in agreement with those from Ferreira da Silva^20^, Cavalcanti et al.^28^, Grandi^18^ and León^21^, who also found an increase in bone neogenesis considering bone filling material in relation to the control cavities, using *software* for image analysis and later morphometric studies.

As we quantitatively analyze the mean values of newly formed bone trabeculae, according to the observation periods, we noticed that the animal model utilized in the study had higher rates of bone formation at 60 days, with homogeneous and superior bone neogenesis values among the test groups submitted to the different bone filling materials tested and the negative control group, with a statistically significant association. At 90 days of the experiment, we noticed a drop in osteogenesis levels in the test cavities from the different materials tested. Throughout all the observation periods and considering the different materials tested, we noticed a larger area of bone neogenesis in the test cavities when compared to their control counterparts - which were filled out only by the blood clot from the animal. This mild reduction in bone neogenesis area at 90 days in all the test cavities from the different materials utilized, suggested that the bone repair process was within a degree of bone maturing progression, which is in agreement with the studied led by Kurashina et al.^29^, who stated that the observation period progression does not cause area size increase, but rather in the bone trabeculate maturation degree.

As to the behavior of the bone surrogates in relation to bone neogenesis, we noticed that the areas of bone trabeculate newly formed by the hydroxyapatite biphasic ceramic were similar and larger than those newly formed by the autogenous bone graft at 60 and 90 days, respectively, showing that such materials are favorable to osteogenic cells, which is in agreement with the studies led by Grandi^18^ and Tsai et al.^30^ and disagrees with those from Cavalcanti et al.^28^, which state that autogenous bone grafts yield better bone neogenesis.

When we studied the chemical makeup of the bioceramic utilized, we could see that that the calcium phosphate cement and the biphasic hydroxyapatite ceramic particles were well differentiated as to particle size, expressing larger hydroxyapatite biphasic ceramic particles, with diameters varying between 0.5 and 0.75mm. The calcium phosphate cement was used as a powder, being converted into a paste after manipulation. The increase in crystal size may influence its maintenance period within the bone defect, with direct impact on the bone neogenesis modulation, considering the large osteoconduction potential presented by the biphasic hydroxyapatite ceramic, following manufacturer's instructions. Thus, one can justify a greater bone trabeculate neogenesis, according to morphometric standards, in the test group of the animals submitted to the biphasic hydroxyapatite ceramic, in all observation periods, when compared to the calcium phosphate cement.

In the morphometric results obtained from the control and test cavities, from the different materials tested, we noticed a statistically significant association in three situations only: at 30 days in the autogenous bone graft group and at 60 days for the calcium phosphate and biphasic hydroxyapatite ceramic. We noticed that the morphometric data from each study group did not follow a homogeneous distribution as far as bone neogenesis is concerned, and there were major differences between bone neogenesis mean values among animals from the same group, thus the non-parametric inference for data evaluation using the median value for inferential assessments. Such behavior variability between animals from the same group can be justified with the studies led by Garcia & Albergaria-Barbosa^13^, which advocated that the grafts may suffer changes during the demineralization and freeze-drying processes, and there is the possible failure in standardization considering the laboratorial preparation of the grafts utilized, which may all be considered factors leading to the different results found between animals of the same group.

It is known that the calcium phosphate cement acts as an osteoconduction biomaterial which in its pure form is converted into hydroxyapatite cement, with the same properties found in the intact bone. It is in this hydroxyapatite conversion process, in which the crystals remain trapped inside the tissue, that osteoconduction is fostered^5,^^6,^^7,^^9,^^10,^^31^. The biphasic hydroxyapatite ceramic behaves in a very different way, promoting neogenesis of bone cells, while the autogenous bone works as osteoinduction, osteoconduction and osteoprogenitor^10,^^29^. Having the morphometric results obtained from the sample studied, we can state that both the calcium phosphate cement as well as the biphasic hydroxyapatite ceramic behaves as osteoconduction materials, since the particles from these materials enable the creation of a framework for the deposition of the osteoinduction cells present in the body.

Based on the morphometric results obtained and linking them to the chemical makeup of the bioceramic, it is believed that the calcium phosphate cement and the biphasic hydroxyapatite ceramic have the necessary characteristics for clinical use. Nonetheless, it is important to understand the goal intended with the application of each one of these materials. In cases when more bone volume is needed, with greater demands concerning bone quality, i.e. correction of defects impacting cosmetics, the biphasic calcium phosphate ceramic can be indicated, given the lingering of its particles, providing the necessary osteoconduction for the body's osteoprogenitor cells, having seen the increase in biphasic hydroxyapatite ceramic particles which enables a greater framework time for the aforementioned cells. For the cases in which goal is the early neogenesis of bone tissue only, i.e. grafting prior to dental implants, the calcium phosphate cement offers faster bone neogenesis with material resorption.

The future of bone surrogates must involve resorbable biological implants, developed with a porous, tridimensional framework, made from resorbable material, filled with morphogenetic proteins and osteoprogenitor cells. A time-dependent controlled resorption of the framework would release the bioactive factors trapped inside its structures, which would induce transplanted and host cells to grow in this intertwining tridimensional pattern. Bone would be formed through the framework and not on the surface only^19^.

## CONCLUSION

Based on the results achieved, we can vouch for the superior bone neogenesis promoted by the biphasic hydroxyapatite ceramic and the calcium phosphate cement when compared to the autogenous bone graft, and such fact places the bioceramics as surgical aides in maxillofacial procedures which require bone grafts.
